# Large Pore Mesoporous Silica and Organosilica Nanoparticles for Pepstatin A Delivery in Breast Cancer Cells

**DOI:** 10.3390/molecules24020332

**Published:** 2019-01-17

**Authors:** Saher Rahmani, Jelena Budimir, Mylene Sejalon, Morgane Daurat, Dina Aggad, Eric Vives, Laurence Raehm, Marcel Garcia, Laure Lichon, Magali Gary-Bobo, Jean-Olivier Durand, Clarence Charnay

**Affiliations:** 1 Institut Charles Gerhardt Montpellier, UMR-5253 Univ Montpellier, CNRS, ENSCM, cc 1701, Place Eugène Bataillon, CEDEX 5, 34095 Montpellier, France; rahmeni.sahar@yahoo.fr (S.R.); jelena.budimir@uni-goettingen.de (J.B.); mylene.sejalon@enscm.fr (M.S.); laurence.raehm@univ-montp2.fr (L.R.); durand@univ-montp2.fr (J.-O.D.); 2 Institut des Biomolécules Max Mousseron, UMR 5247 CNRS, UM-Faculté de Pharmacie, 15 Avenue Charles Flahault, CEDEX 5, 34093 Montpellier, France; morgane.daurat2@gmail.com (M.D.); dina.aggad@umontpellier.fr (D.A.); laure.lichon@umontpellier.fr (L.L.); 3 NanoMedSyn, Faculté de Pharmacie, 15 Avenue Charles Flahault, CEDEX 5, 34093, Montpellier, France; 4 Centre de Recherche en Biologie cellulaire de Montpellier (CRBM), UMR 5237 CNRS, Université Montpellier, 1919 Route de Mende, CEDEX 5, 34293 Montpellier, France; eric.vives@umontpellier.fr (E.V.); marcel.garcia@inserm.fr (M.G.)

**Keywords:** pepstatin A, mesoporous silica nanoparticles, mesoporous organosilica nanoparticles, cancer

## Abstract

(1) Background: Nanomedicine has recently emerged as a new area of research, particularly to fight cancer. In this field, we were interested in the vectorization of pepstatin A, a peptide which does not cross cell membranes, but which is a potent inhibitor of cathepsin D, an aspartic protease particularly overexpressed in breast cancer. (2) Methods: We studied two kinds of nanoparticles. For pepstatin A delivery, mesoporous silica nanoparticles with large pores (LPMSNs) and hollow organosilica nanoparticles (HOSNPs) obtained through the sol–gel procedure were used. The nanoparticles were loaded with pepstatin A, and then the nanoparticles were incubated with cancer cells. (3) Results: LPMSNs were monodisperse with 100 nm diameter. HOSNPs were more polydisperse with diameters below 100 nm. Good loading capacities were obtained for both types of nanoparticles. The nanoparticles were endocytosed in cancer cells, and HOSNPs led to the best results for cancer cell killing. (4) Conclusions: Mesoporous silica-based nanoparticles with large pores or cavities are promising for nanomedicine applications with peptides.

## 1. Introduction

Mesoporous silica nanoparticles (MSNs) hold great promise for biological applications, particularly in the field of theranostics and drug delivery, and the field has been extensively reviewed [1,2,3,4,5,6,7,8,9,10,11,12,13,14,15]. Indeed, these nanoparticles (NPs) exhibit adjustable diameters (10 to 200 nm) and pore size (2–15 nm), leading to wide ranges of encapsulated drugs and biomolecules. The synthesis of these NPs, especially those presenting a large pore and which are targeted, has recently been performed [16] and both methods for their preparation and colloidal stability studies have been reviewed [17]. Large-pore mesoporous silica nanoparticles (LPMSNs) are of interest for the delivery of large molecules, such as nucleic acids or peptides [18]. The release and delivery of peptides for antibacterial [19,20] and anticancer applications has been recently reported [21,22,23]. Although less described than MSNs, organosilica NPs [24,25] and their porous systems [26,27,28] are very promising for bioapplications. The properties of organosilica NPs are very different from those of MSNs in terms of stability, drug loading, and release capacities, due to the high content of organic groups, which constitute the structure [29,30]. Recently, large-pore mesoporous organosilica NPs have been synthesized for the delivery of therapeutic proteins in cells [31]. LPMSNs and organosilica NPs seem good candidates for the delivery of pepstatin A (C_34_H_63_N_5_O_9_), a small hydrophobic pentapeptide which is the most potent inhibitor of cathepsin D, a lysosomal aspartic endopeptidase overexpressed in solid tumors and breast cancer [32]. Overexpression of cathepsin D is associated with tumor growth. Therefore, an efficient system which would inhibit cathepsin D could be of high interest for cancer therapy. As pepstatin A does not cross cell membranes, it has to be vectorized in cancer cells; nevertheless, very few studies report the vectorization of pepstatin A with NPs. One study used pepstatin A covalently linked to superparamagnetic iron oxide NPs to target P-glycoproteins in the brain of epilepsy rats [33]. In this work, we present the syntheses of LPMSNs functionalized with fluorescein isothiocyanate (FITC) and of new tertiary amine-based hollow organosilica nanoparticles (HOSNPs) obtained with a pore expanding agent. Then, their noncovalent loading was investigated with two short peptides, pepstatin A, and a model cyclic protected Arg-Gly-Asp (RGD) peptide whose adsorption and release can be monitored by UV-visible spectroscopy, unlike pepstatin A. The endocytosis of these NPs was monitored in MCF-7 breast cancer cells, and delivery of pepstatin A was successfully demonstrated in MCF-7 breast cancer cells.

## 2. Results and Discussion

**Figure 1:**
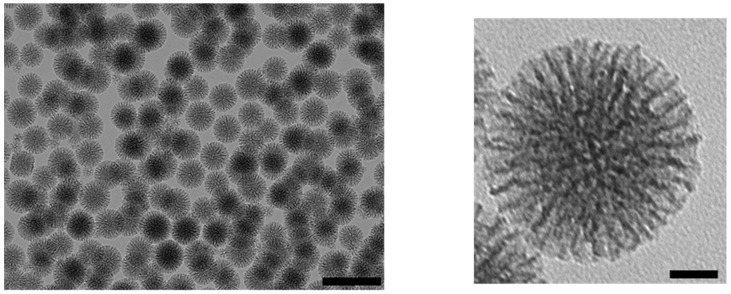
TEM image of large-pore mesoporous silica nanoparticles (LPMSNs). Scale Bar 200 nm (**left**) and 50 nm (**right**), respectively.

The synthesis of LPMSNs was performed as already described [16]. Monodispersed NPs with 100 nm diameter were observed by transmission electron microscopy (TEM) images and had 7 nm in pore diameter radial mesopores (Figure 1). Although some spontaneous nuclei were observed, incorporation of fluorescein isothiocyanate (FITC) linked to aminopropyltriethoxysilane (APTES) in the walls did not modify the mesostructure. N_2_ adsorption–desorption measurements showed type IV isotherms and the specific surface area was 817 m^2^·g^−1^. The pore size distribution shows that LPMSNs had 3-nm- and 7-nm-sized pores (Supplementary Figure S1). Zeta potential was negative at pH 7.4 (−19.5 mV), which is consistent with the deprotonation of the surface silanol groups.

**Figure 2:**
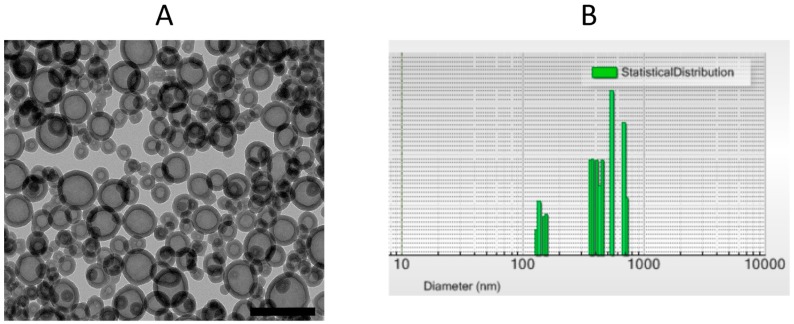
(**A**) TEM image of hollow organosilica nanoparticles (HOSNPs). Scale Bar 100 nm. (**B**) Dynamic light scattering in intensity mode of HOSNPs (dispersed in EtOH).

HOSNPs were synthesized by hydrolysis and polycondensation of bis (3-tri-methoxysilyl)-*N*-methylamine (BMSPMA) at 75 °C in basic media (pH = 10) using cetyltrimethylammonium bromide (CTAB) as the template and 1,3,5-triethylbenzene (TEB) as the pore-expanding agent. The surfactant was extracted with a solution of ammonium nitrate in ethanol. TEM images showed a hollow structure with small NPs of 20–30 nm diameter and larger NPs with a mean diameter of 100 nm and with 10–15 nm thick walls (Figure 2A). Dynamic light scattering in EtOH confirmed two populations of HOSNPs with mean hydrodynamic diameters of 228 nm and 460 nm, respectively (Figure 2B), in agreement with the TEM observations; however, some aggregates were observed with hydrodynamic diameters around 900 nm. HOSNPs were further characterized by solid-state ^29^Si and ^13^C cross-polarization magic angle spinning nuclear magnetic resonance (CP/MAS NMR) spectroscopy (Figure S2). ^29^Si CP/MAS NMR spectrum (Figure S2A) showed signals at −64, −72, and −82 ppm, corresponding to T^1^, T^2^, and T^3^ units (T*^m^*: C*Si*(OSi)*_m_*(OH)_3−*m*_), respectively, in agreement with a well-condensed structure. ^13^C CP/MAS NMR spectrum (Figure S2B) clearly displayed three signals at 10, 21, and 43 ppm, corresponding to the propyl chains of condensed BMSPMA and the signal of the methyl group linked to nitrogen was found at 64 ppm. In addition, XRD patterns at small angles (Figure S3A) displayed the presence of a diffraction peak at around 1.9°, which suggests porosity in the walls. In addition, the wide angle XRD of HOSNPs (Figure S3B) showed a broad peak at 23° characteristic of nonregular repetitions within the siloxane framework. N_2_ adsorption–desorption isotherm showed a specific surface area of 58 m^2^·g^−1^ with a pore size of 26 nm. The type II isotherm (IUPAC) is consistent with nonporous materials at 77 K with adsorption–desorption of nitrogen in interparticle voids, which suggests that the large cavity is not accessible to nitrogen at cryogenic temperature of 77 K (Figure S4). Zeta potential was positive at pH 7.4 (+32 mV), in agreement with the protonation of tertiary amine group.

**Figure 3:**
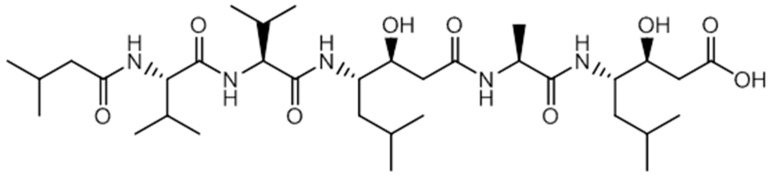
Chemical structure of the pepstatin A (C_34_H_63_N_5_O_9_, Mw = 686 g·mol^−1^).

We then examined the prepared NPs for the vectorization of peptides. First, a model cyclic protected RGD peptide was loaded in HOSNPs in water under stirring for 24 h. After centrifugation for 20 min and washes with water, the NPs were dried under vacuum. The loading capacity of the HOSNPs with the RGD peptide was 28 wt% (Figure S6). The release of the RGD peptide was analyzed in water at pH 7 (Figure S7). No release was observed in these conditions. We observed 16 wt% peptide release after adjusting the pH to 5. This experiment shows the versatility of HOSNPs for the adsorption/release of different short peptides. The prepared LPMSNs and HOSNPs were then loaded with hydrophobic pepstatin A (Figure 3) in EtOH, under stirring for 48 h. After centrifugation, the NPs were washed with EtOH, dried under vacuum, and stored at 0 °C. The dosage of the supernatants with HPLC/MS allowed to determine the loading capacities of the NPs: 18 wt% for HOSNPs and 32 wt% for LPMSNs, respectively. After loading of pepstatin A, the zeta potential of LPMSNs decreased from −19.5 mV to −22.6 mV, whereas the zeta potential of HOSNPs decreased from +32 mV to −13.2 mV. This latest high variation of zeta potential suggests that pepstatin A was mainly adsorbed onto the external surface of HOSNPs, whereas hydrophobic pepstatin A was mainly adsorbed into the pores of LPMSNs.

Pepstatin A release was then investigated. The loaded NPs were placed at the bottom of a cuvette that was carefully filled with water to avoid the nanoparticle swirling. Pepstatin A release from the NPs was monitored with the analysis of the solution by HPLC/MS. No significant release of pepstatin A from the HOSNPs was observed and a low release from LPMSNs was noticed over time (Figure S5A,B). The strong electrostatic interactions between negatively charged pepstatin A and the ammonium moieties of HOSNPs prevented the release of the peptide.

**Figure 4:**
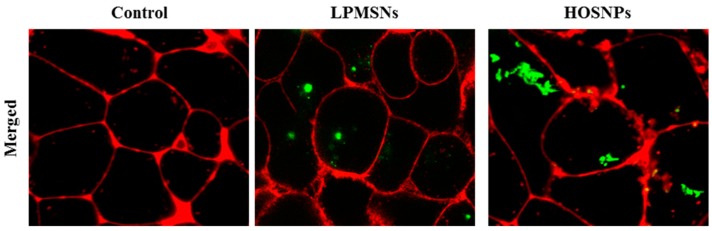
Fluorescence confocal imaging of live MCF-7 cells incubated for 24 h with LPMSNs or HOSNPs. The presence of the NPs is marked by green dots and membranes of cells appear in red.

The endocytosis of the NPs was then investigated with MCF-7 breast cancer cells. HOSNPs were loaded with FITC in order to monitor the uptake of the NPs in the cells by confocal microscopy. LPMSNs (already functionalized with FITC) and loaded HOSNPs were incubated for 24 h with MCF-7 cells at a concentration of 50 µg·mL^−1^. The membranes of the cells were stained with a cell mask 15 min before observation (Figure 4). After 24 h of incubation, the presence of the NPs in the cytoplasm of the cells was clearly observed, showing the endocytosis of LPMSNs and HOSNPs.

**Figure 5:**
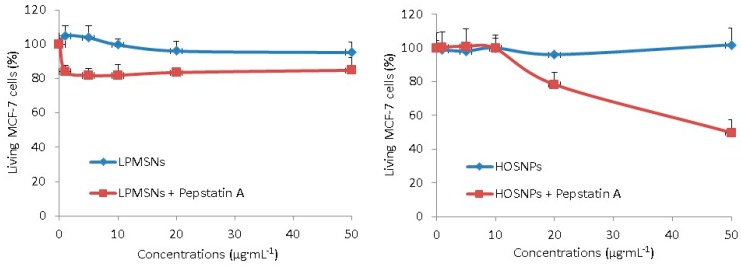
Cytotoxic study of LPMSNs and HOSNPs loaded or not with pepstatin A. Human breast cancer cells (MCF-7) were incubated during 72 h with increasing concentrations (from 1 to 50 µg·mL^−1^) of NPs loaded or not with pepstatin A. Values are means ± standard deviations of 3 experiments.

The delivery of pepstatin A was investigated in MCF-7 cancer cells, as pepstatin A does not cross cell membranes and the peptide has to be encapsulated to be efficiently vectorized. The pepstatin A-loaded NPs or unloaded NPs were incubated with MCF-7 cancer cells for 72 h at several concentrations and MTT assay allowed to determine cancer cell death. The unloaded NPs were not toxic, even with HOSNPs, which have a positive zeta potential. These ammonium-based NPs were thus biocompatible. When loaded with pepstatin A, LPMSNs led to 20% of cancer cell death, whereas up to 60% cancer cell death was observed with HOSNPs (Figure 5). As shown by pepstatin A release monitoring, LPMSNs slightly released pepstatin A, whereas no release was noticed for HOSNPs. Although the loading capacity was higher with LPMSNs, we assume that premature release occurred with LPMSNs before endocytosis when incubated with cancer cells. More sustained delivery of pepstatin A in cells with HOSNPs could explain the results, due to stronger interactions between HOSNPs and pepstatin A than with LPMSNs.

## 3. Materials and Methods

Cetyltrimethylammonium bromide (CTAB), ammonium hydroxide, ammonium nitrate (NH_4_NO_3_), potassium bromide, 1,3,5-triethylbenzene (TEB), and pepstatin A were purchased from Sigma-Aldrich (Saint-Quentin-Fallavier, France). Absolute ethanol was purchased from Fisher Chemicals. Bis (3-trimethoxysilyl propyl)-*N*-methylamine (BMSPMA) was purchased from Abcr GmbH&Co (Karlsruhe, Germany) and hydrochloric acid from VWR PROLABO. Triethanolamine (TEA) as a base catalyst, and acetic acid were purchased from Wako Pure Chem. Ind., Ltd (Osaka, Japan). Tetraethoxysilane (TEOS: Si(OC_2_H_5_)_4_), tetrapropoxysilane (TPOS: Si(OC_3_H_7_)_4_), 3-aminopropyltriethoxysilane (APTES), and fluorescein 5-isothiocyanate (FITC) were purchased from Tokyo Chemical Industry Co., Ltd (Osaka, Japan).

The complete characterization of the synthesized NPs requires a complementary panel of techniques. In this study, techniques focusing on the properties of NPs, such as transmission electron microscopy (TEM), dynamic light scattering (DLS), X-ray diffraction (XRD), and nitrogen adsorption–desorption were implemented. TEM images were recorded with a JEOL 1200 EXII microscope to visualize the shape and size of the NPs. For the purpose of TEM analysis, the sample particles were dispersed in ethanol and then dropped onto copper grids with porous carbon films. Dynamic light scattering analyses were performed using a Cordouan Technologies DL 135 particle size analyzer instrument. For zeta potential, the suspensions were prepared by dilution in NaCl (1 mM) in order to impose the ionic strength and analysis was performed using a Malvern Zetasizer nano ZS (ZEN 3600) instrument. The zeta potential was calculated from the measured electrophoretic mobility based on the theoretical analysis related to the Helmholtz–Smoluchowski equation. [34] For ^13^C CP/MAS NMR spectra, solid-state NMR experiments were performed on a Varian VNMRS 600 MHz (14.1 T) NMR spectrometer. The specific area and pore structure parameters of the studied NPs were determined from the measurements of nitrogen adsorption–desorption at 77 K with a TRISTAR 3000 Micromeritics using the Brunauer–Emmet–Teller (BET) method. For the sorption experiments, the samples (about 70 mg) were evacuated under vacuum at 80 °C for 12 h. The organization of the porous framework was controlled by XRD and performed with a PANalytical X’Pert MPD (Philips 1710) diffractometer. The small angles measurements (2θ from 1.5 to 10) were recorded with an adapted slit of 1/16.

### 3.1. Synthesis of HOSNPs

A total of 20 mg of CTAB (5.5 × 10^−5^ mol) and 1.5 mL of TEB (8 × 10^−3^ mol) were mixed in 60 mL of water and 300 μL of ethanol and stirred for one night at 75 °C/1000 rpm. A total of 40 μL of NaOH solution at 1 M (4 × 10^−5^ mol) was then added and the mixture vigorously stirred for 50 min at 75 °C/1000 rpm. Then, 156 μL (0.44 × 10^−3^ mol) of BMSPMA was added. The reaction was stirred for 2 h at 80 °C/1400 rpm. The reaction mixture was cooled to room temperature and centrifuged for 15 min, 20,000 rpm. Surfactants were removed by three extractions, with 30 mL of a solution of ammonium nitrate (NH_4_NO_3_) in ethanol (0.075 M), followed by three ethanol washes (20 mL each). NPs were dried under vacuum at room temperature.

### 3.2. Synthesis of LPMSNs

Triethanolamine (4.7 × 10^−3^ mol) and 3.33 g of CTAB (9.1 × 10^−3^ mol) were dissolved in 400 mL of deionized water in a round-bottomed flask (500 mL), and the mixture was stirred at 900 rpm for 1 h at 80 °C using a magnetic stirrer with a football-type stirring bar. Then, 0.8 mL of tetraethoxysilane (3.6 × 10^−3^ mol) was added to the solution with stirring at 900 rpm. After stirring the mixture at 80 °C for 1 h, the obtained colloidal solution was cooled to room temperature with stirring. Then, ethanol containing FITC-APTES and 1,3,5-triisopropylbenzene (1.8 × 10^−3^ mol) was added to the obtained colloidal solution (200 mL). FITC–APTES was synthesized by adding FITC (2.2 × 10^−5^ mol) and APTES (8 × 10^−8^ mol) into the ethanol (2.5 mL) and stirring the mixture in a dark and cool place overnight. Then, tetrapropoxysilane (TPOS) (4 × 10^−3^ mol) was added into the colloidal solution, and the colloidal solution was stirred for 2 days at room temperature. The process of the addition of TPOS was repeated up to four times. The prepared colloidal solution (50 mL) was transferred into a dialysis membrane tube composed of cellulose (molecular weight cutoff 12,000–14,000 Da) and was dialyzed for 24 h against a mixture (250 mL) of 2 M aqueous acetic acid and 2-propanol (1:1, *v*/*v*) to remove organic species. This process was repeated five times. After that, the colloidal solution was dialyzed for 24 h against water (250 mL), and this process was repeated four times.

### 3.3. Loading of Nanoparticles with Pepstatin A

A total of 10 mg of NPs and 5 mg of pepstatin A in 4 mL of ethanol were sonicated (45 kHz) for 5 min and stirred for 48 h at room temperature at 320 rpm. Then, the NPs were centrifuged at 12,000 rpm for 25 min, washed 2 times with ethanol (1 mL each), and dried for five hours under vacuum.

The loading capacity was calculated through the following formula: Loading Capacity (wt%) = [(initial mass of loaded pepstatin − mass of unloaded pepstatin A)/mass of NPs obtained + mass of loaded pepstatin A] × 100. The mass of unloaded pepstatin was calculated from the titration of the supernatant of pepstatin A in a solution with HPLC/MS. The loading capacity was 18 wt% for HOSNPs and 32 wt% for LPMSNs.

### 3.4. Loading of HOSNPs with Protected RGD Peptide

A total of 5 mg of HOSNPs was sonicated (45 kHz) for 10 min in 5 mL of deionized water. A total of 3.9 mg of peptide was then added to the mixture. The solution was stirred at 320 rpm at room temperature for 24 h. Finally, the NPs were collected by centrifugation for 12 min at 12,000 rpm, washed three times with water (2 mL each), and dried for five hours under vacuum. A total of 3.6 mg of loaded NPs was obtained. UV-visible allowed to determine the loading capacity of the HOSNPs: 28 wt%.

### 3.5. Loading of HOSNPs with Fluorescein Isothiocyanate (FITC)

First, 10 mg of NPs was added in an ethanol solution (4 mL) of fluorescein isothiocyanate (FITC, 7 µmol) and stirred for 48 h at room temperature. The NPs were collected by centrifugation for 12 min at 12,000 rpm, washed twice with ethanol (2 mL each), and dried overnight under air.

### 3.6. Cell Culture

Human breast cancer cells MCF-7 were purchased from ATCC (American Type Culture Collection, Manassas, VA, USA). Cells were cultured in Dulbecco’s Modified Eagle’s Medium (DMEM-F12) supplemented with 10% fetal bovine serum and 50 μg·mL^−1^ gentamycin. These cells were allowed to grow in a humidified atmosphere at 37 °C under 5% CO_2_.

### 3.7. Fluorescence Imaging of Cell Uptake of NPs

Human breast cancer cells MCF-7 were seeded into a tissue culture chamber with a cover glass bottom in 300 µL of culture medium. The culture medium is based on a Dulbecco’s Modified Eagle Medium: Nutrient Mixture F-12 (DMEM/F12) that is a commercial medium intensively used for the culture of mammalian cells and available in all consumable providers for cell biology. Moreover, 10% fetal bovine serum was added for proteins, fatty acids, and growth factor addition. To finish, this medium was supplemented with antibiotics to assure cell safety. Then, the cells were incubated for 24 h with fluorescein functionalized NPs at a concentration of 50 µg·mL^−1^. After incubation, the cells were washed twice with 1 mL of culture medium. Fluorescence imaging (488 nm excitation wavelength) was performed on living cells with a Carl Zeiss confocal microscope.

### 3.8. Cytotoxic Study

MCF-7 cells were seeded into 96-well plates at 500 cells per well in 200 μL of the previously described culture medium and allowed to grow for 24 h. Increasing concentrations of NPs with or without pepstatin A were added in a culture medium of MCF-7 cells. Three days after treatment, an MTT assay was performed to determine the drug delivery potential of the NPs. Briefly, cells were incubated for 4 h with 0.5 mg·mL^−1^ of MTT (3-(4,5-dimethylthiazol-2-yl)-2,5-diphenyltetrazolium bromide; Promega) in media. The MTT/media solution was then removed, and the precipitated crystals were dissolved in 150 µL of EtOH/DMSO (*v*/*v*). The solution absorbance was read at 540 nm in a microplate reader.

## 4. Conclusions

The syntheses of LPMSNs or HOSNPs were successfully achieved through the sol–gel procedure in mild conditions, and the NPs were loaded with pepstatin A, a hydrophobic peptide inhibitor of cathepsin D. The loading capacities of the NPs were high with at 32 wt% and 18 wt%, respectively. After incubation with MCF-7 breast cancer cells, the NPs (50 µg·mL^−1^) were endocytosed as monitored by fluorescence confocal microscopy. LPMSNs and HOSNPs loaded with pepstatin A and incubated with cancer cells led to significant 20% and 60% cancer cell death, respectively. We assume that electrostatic interactions between pepstatin A and HOSNPs were stronger than the interactions between LPMSNs and pepstatin A, which could explain the better activity of HOSNPs through a more sustained delivery of pepstatin A in cells. Organosilica-based nanoparticles thus represent an interesting tool for the vectorization of peptides for nanomedicine applications.

## Supplementary Materials

The following are available online at http://www.mdpi.com/1420-3049/24/2/332/s1, Figure S1: N_2_ adsorption desorption isotherm, BET and BJH of LPMSN, Figure S2: ^29^Si (A) and ^13^C CP/MAS (B) Solid-state NMR of HOSNPs Figure S3: XRD patterns at small angles (A) and wide angles (B). Figure S4: N_2_ adsorption desorption, BET and BJH of HOSNPs. Figure S5: Release of pepstatin A from HOSNPs and LPMSNs monitored with HPLC/MS, Figure S6: Structure of the RGD peptide and UV-Vis analysis in water, Figure S7: Release of the RGD peptide from HOSNPs at pH 7 and pH 5.
