# Supplementary material for: Large Pore Mesoporous Silica and Organosilica Nanoparticles for Pepstatin A Delivery in Breast Cancer Cells
Source: Molecules. 2019 Jan 17;24(2):332. doi: 10.3390/molecules24020332 (PMC6359328; doi:10.3390/molecules24020332)
Supplement: Supplementary file 1 [file molecules-24-00332-s001.pdf]

Supplementary Materials:

Large Pore Mesoporous Silica and Organosilica Nanoparticles for Pepstatin A Delivery in Cancer Cells.

Saher Rahmani [1], Jelena Budimir [1], Mylene Sejalon [1], Morgane Daurat [2,3], Dina Aggad [2], Eric Vivès [4], Laurence Raehm [1], Marcel Garcia [4], Laure Lichon [2], Magali Gary-Bobo [2,*], Jean-Olivier Durand [1] and Clarence Charnay [1,*]

[1] Institut Charles Gerhardt Montpellier, UMR-5253, Univ Montpellier, CNRS, ENSCM, cc 1701, Place Eugène Bataillon, Cedex 5, 34095 Montpellier, France; rahmeni.sahar@yahoo.fr (S.R.); jelena.budimir@uni-goettingen.de (J.B.); mylene.sejalon@enscm.fr (M.S.); laurence.raehm@univ-montp2.fr (L.R.); durand@univ-montp2.fr (J.-O.D.)

[2] Institut des Biomolécules Max Mousseron, UMR 5247 CNRS, UM-Faculté de Pharmacie, 15 Avenue Charles Flahault, Cedex 5, 34093 Montpellier, France; morgane.daurat2@gmail.com (M.D.); dina.aggad@umontpellier.fr (D.A.); laure.lichon@umontpellier.fr (L.L.)

[3] NanoMedSyn, Faculté de Pharmacie, 15 Avenue Charles Flahault, Cedex 5, 34093, Montpellier, France

[4] Centre de Recherche en Biologie cellulaire de Montpellier (CRBM), UMR 5237 CNRS, Université Montpellier, 1919 Route de Mende, Cedex 5, 34293 Montpellier, France; eric.vives@umontpellier.fr (E.V.); marcel.garcia@inserm.fr (M.G.)

* Correspondence: magali.gary-bobo@inserm.fr (M.G.-B.); clarence.charnay@umontpellier.fr (C.C.); Tel.: C.C. +33-467-14-38-64

Supplementary Figures:

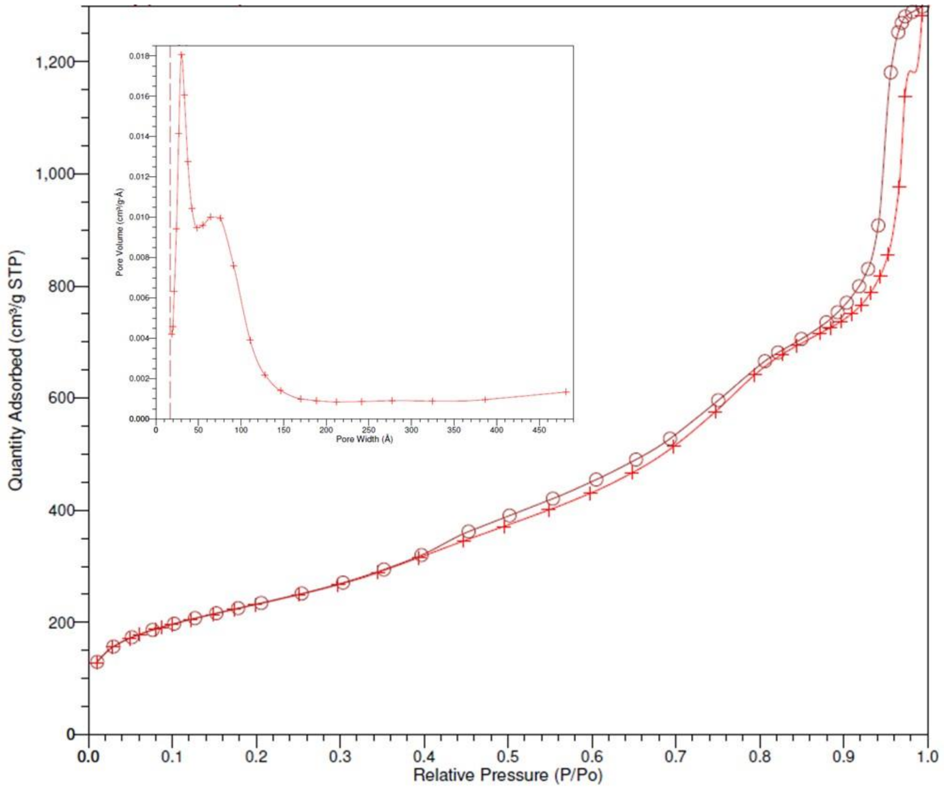

Figure S1. N_2 adsorption-desorption isotherm of LPMSNs and pore size distribution calculated by BJH method using the desorption branch. BJH adsorption cumulative volume of pores was 1.45 cm³/g, and BJH desorption cumulative volume of pores was 1.93 cm³/g. Although 3 nm sized pores were observed

in the pore size distribution, this should be partly due to the cavitation of nitrogen from the pores in the core part of LPMSNs.

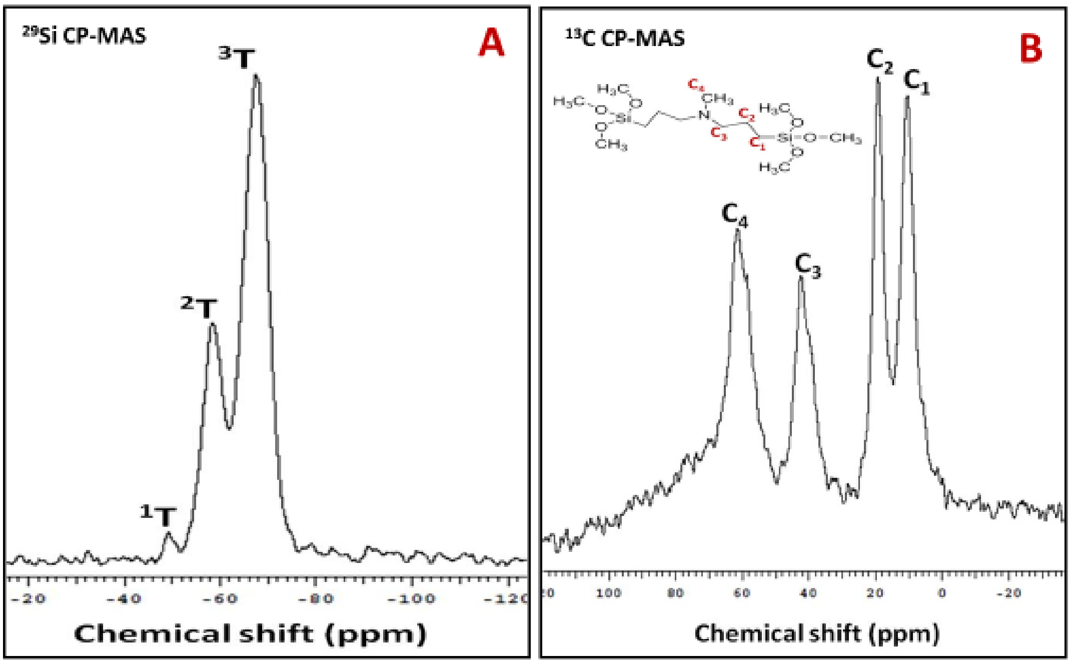

Figure S2. ^{29}Si (A) and ^{13}C CP/MAS (B) Solid state NMR of HOSNPs.

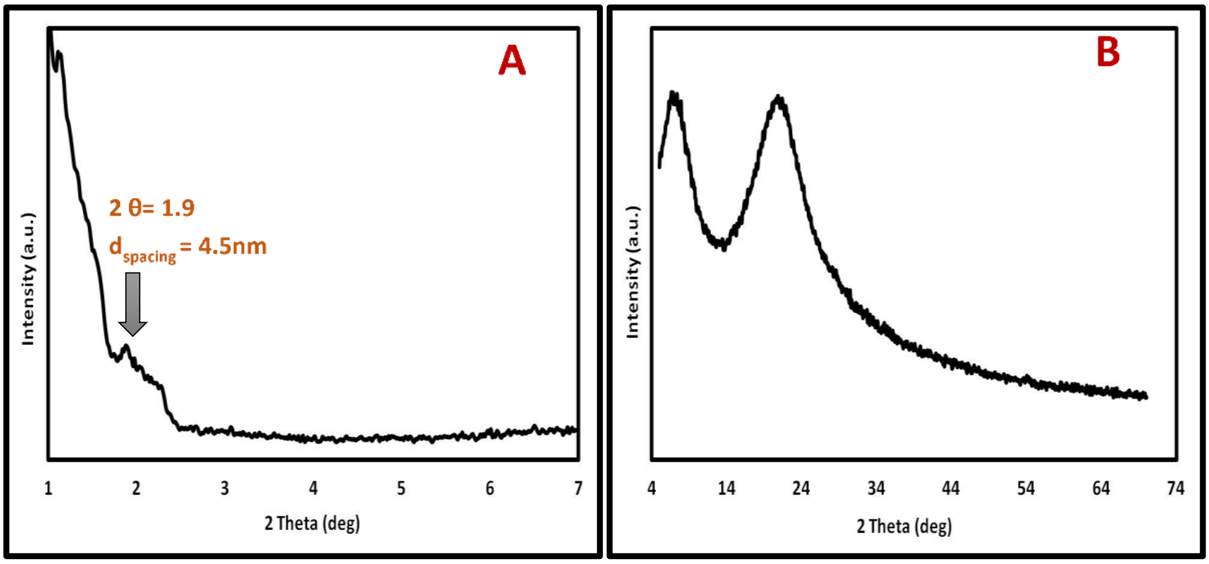

Figure S3. XRD patterns at small angles (A) and wide angles (B)

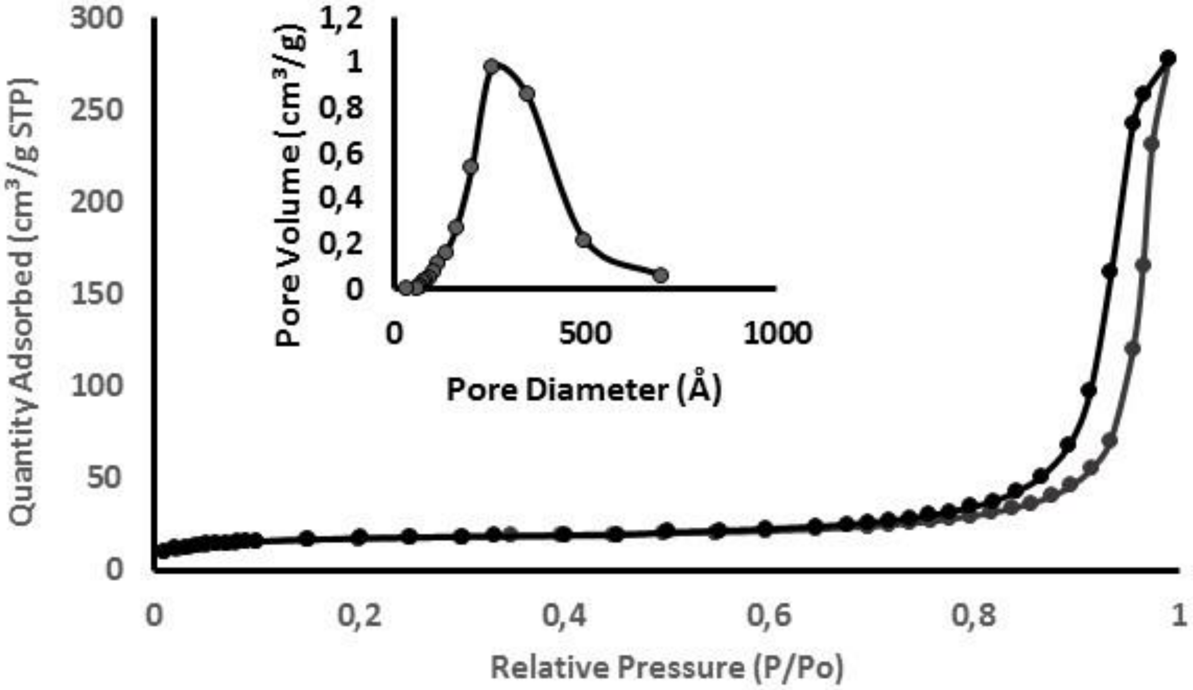

Figure S4. N_2 adsorption desorption, BET and BJH of HOSNPs. BJH Adsorption and Desorption cumulative volume of pores: 0.42 cm³/g.

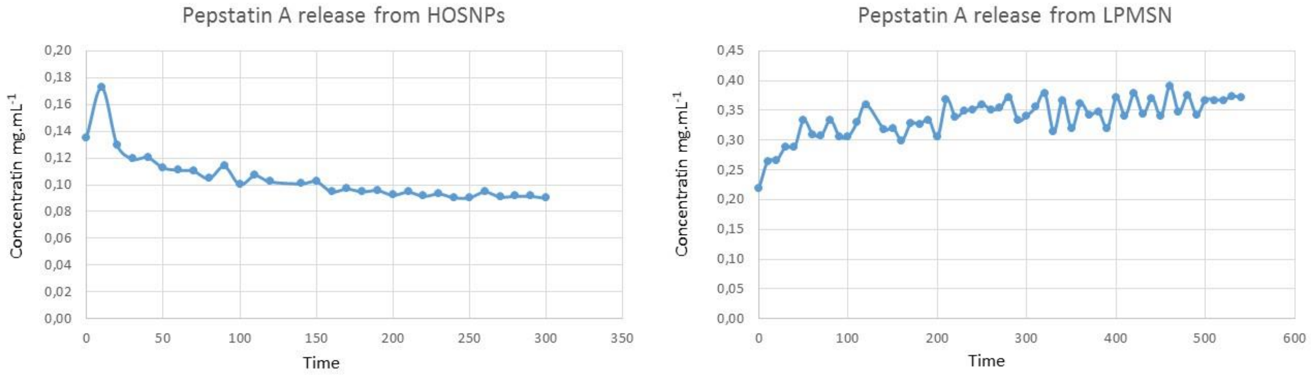

Figure S5. Release of Pepstatin A from HOSNPs and LPMSNs monitored with HPLC/MS.

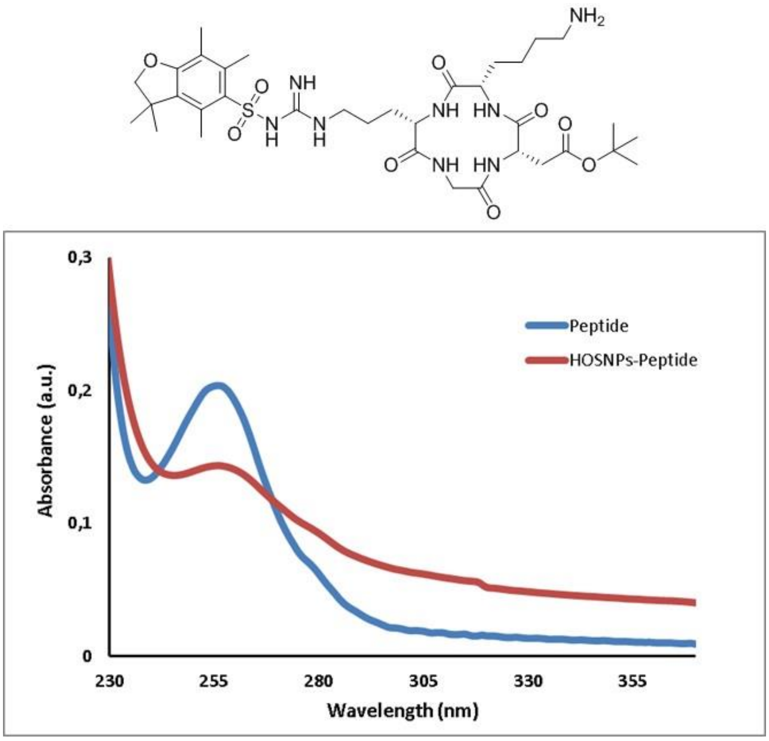

Figure S6. Structure of the RGD peptide and UV-Vis analysis in water.

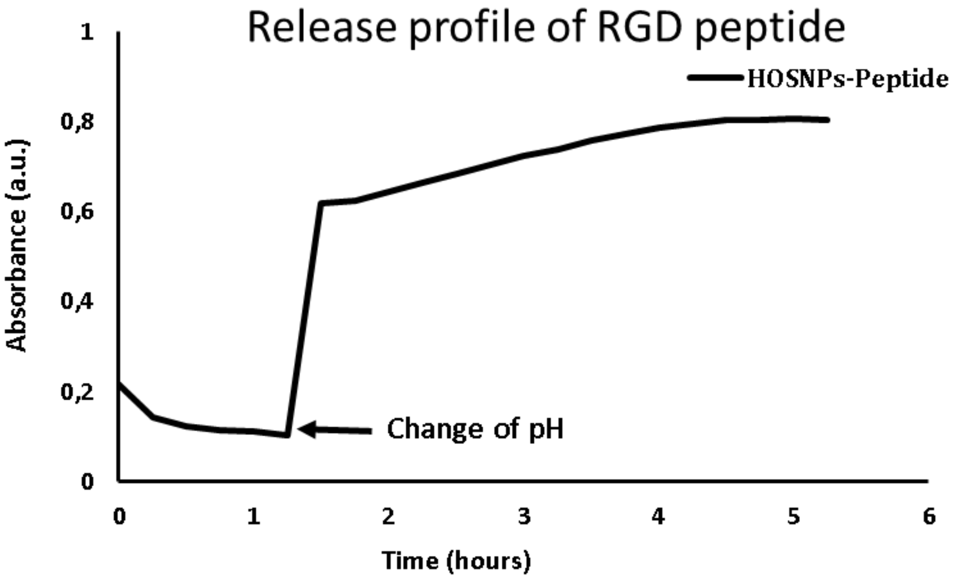

Figure S7. Release of the RGD peptide from HOSNPs at pH 7 and pH 5 monitored at 255 nm, 16% of the peptide was released.
